# Papillary Thyroid Carcinoma as a Lateral Neck Cyst: A Cystic Metastatic Node versus an Ectopic Thyroid Tissue

**DOI:** 10.1155/2018/5198297

**Published:** 2018-10-18

**Authors:** Frhana Rahmat, Ananth Kumar Marutha Muthu, Navarasi S Raja Gopal, Soh Jo Han, Azura Sharena Yahaya

**Affiliations:** ^1^Faculty of Medicine & Defence Health, National Defence University of Malaysia, Sungai Besi Camp, 57000 Kuala Lumpur, Malaysia; ^2^Pathologist, Hospital Angkatan Tentera Tuanku Mizan, Wangsa Maju, KL, Malaysia; ^3^Pathologist, Hospital Putrajaya, Malaysia; ^4^General Surgeon, Hospital Angkatan Tentera Tuanku Mizan, Wangsa Maju, KL, Malaysia; ^5^Radiologist, National Defence University of Malaysia, Malaysia

## Abstract

Papillary thyroid carcinoma is the most common thyroid malignancy and frequently metastasizes to regional lymph nodes. Occasionally, metastatic lymph nodes are palpable without the evidence of primary tumour. Papillary thyroid carcinoma of lateral neck cyst is a rare condition. It may arise from thyroid primary which underwent cystic degeneration or true malignant transformation of ectopic thyroid tissue. Herein, we reported two cases with preoperative diagnosis of benign lateral neck cyst but postoperative histopathological results showed primary papillary thyroid carcinoma. Ultrasonography and computed tomography of the neck in both cases showed no significant thyroid lesion. However, the patient in Case  2 was subjected for total thyroidectomy and histopathological results showed the origin of primary tumour. In conclusion, thorough investigations including total thyroidectomy are indicated in cases of papillary thyroid carcinoma of lateral neck cyst. This practice is to ensure that this type of thyroid cancer can be detected earlier because it has a very good prognosis if treated earlier.

## 1. Introduction

Papillary carcinoma of the thyroid (PTC) is the most common thyroid malignancy accounting for 80% of all thyroid cancers and predominantly occurs in the third and fourth decades of life [1]. It has a predisposition for lymphatic metastasis and occasionally metastatic lymph nodes are palpable without the primary tumour being evident [2]. Although rare, cystic metastasis in the neck lymph node can occur, and papillary thyroid carcinoma may present as a benign lateral neck cyst.

Embryologically, the thyroid gland is derived as an epithelial proliferation in the floor of the pharynx between the tuberculum impar and the copula at a point later indicated by the foramen cecum. Later, the thyroid descends in front of the pharyngeal gut as a bilobed diverticulum. During the migration process, the thyroid remains connected to the tongue by thyroglossal duct which later disappears [3].

Ectopic thyroid gland is defined as thyroid tissue localized outside of the second to fourth tracheal cartilages [4]. It is most commonly found anywhere along the path of descent of the thyroid gland [3]. It is a rare developmental abnormality and the prevalence is approximately 1 per 100,000–300,000 people [5]. However, lateral ectopic thyroid tissue is a much less common condition [6].

Herein, we reported two cases of PTC incidentally found as lateral neck cyst.

## 2. Case Report

### 2.1. Case 1

A 32-year-old man presented with a 2-year history of a painless right-sided neck mass.

**Figure 1:**
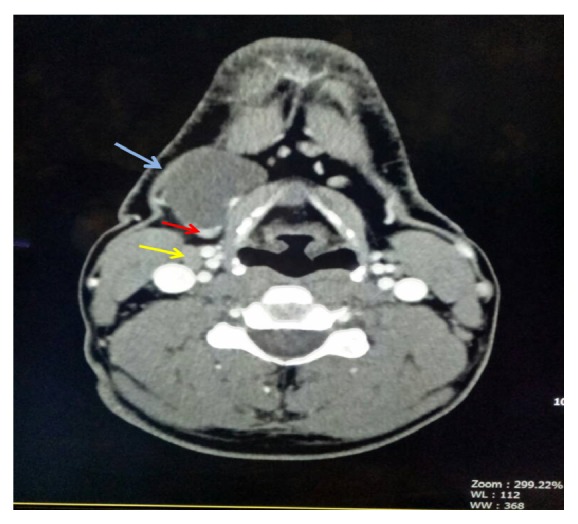
A well-defined cystic lesion (blue arrow) noted at the anteroinferior to the right submandibular gland measuring about 3.0 x 2.0 x 2.2 cm. There is a 0.8 cm enhancing nodule (red arrow) within the lesion. Enlarged submandibular lymph node seen (yellow arrow).

**Figure 2:**
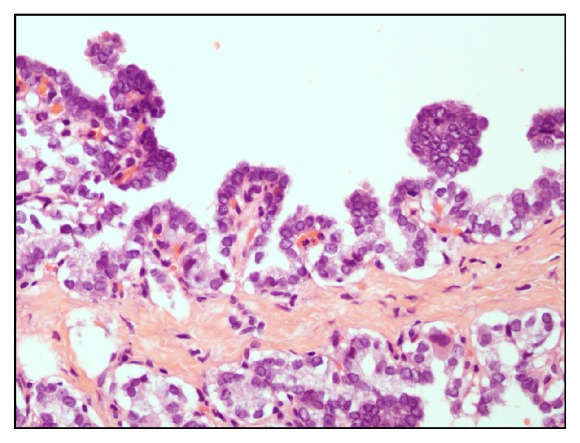
High power (40x) microscopy of the lateral neck mass showing papillary thyroid carcinoma features.

Clinical examination revealed 3 x 3 cm smooth, round, nontender, mobile mass at the right lateral of the neck, at the submandibular region. No palpable lymph node was present. Computed tomography (CT) scan neck region revealed a well-defined 3.0 x 2.2 x 2.0 cm uniloculated lesion located anteroinferior to the right submandibular gland with imperceptible wall. An 8 mm enhancing nodule was noted within the lesion at its inferior pole. Subcentimeter submental and bilateral submandibular lymph nodes are present. Bilateral enlarged cervical lymph nodes about 1 cm size at level II are identified. The thyroid is normal in appearance with no focal lesion seen (Figure 1). He was admitted for further treatment under the impression of infected dermoid cyst. Intraoperatively, there was a cyst measuring 3 x 3 cm located posterior to the strap muscle and lateral to the thyroid cartilage. Excision biopsy of the lateral neck cyst was done. Grossly, the excised specimen revealed a thin-walled cyst containing chocolate-brown fluid, measuring 35 x 30 x 20 mm. The inner surface showed multiple small excrescences ranging from 2 to 3 mm in diameter. Pathologic examination revealed the specimen to be a papillary thyroid carcinoma with a prominent cystic change (Figure 2). Immunohistochemical stain shows the tumour cells are reactive to TTF-1 (nucleus staining). One out of two tiny lymph nodes (2-3 mm in largest diameter) showed tumour metastasis. Postoperative fine needle aspiration (FNA) of both thyroid lobes revealed no evidence of PTC.

### 2.2. Case 2

A 53-year-old male presented with persistent left sided neck swelling for 2 years. Physical examination revealed a cystic mass at the left posterior triangle of the neck measuring 5 x4 cm. The CT scan of neck region revealed well-defined complex cystic lesions with foci of calcifications and small area of enhancement, measuring 4.3 x 3.8 x 5cm and 1.7 x 1.9 cm, located at posterior cervical space and appear to be extending to left supraclavicular region inferiorly. Multiple enhancing subcentimeter bilateral submental and cervical nodes are also seen. The sonographic findings showed homogenous thyroid gland with no focal lesion or abnormal vascularity seen. The thyroid function test is within normal range. Thus, the initial impression was inflammatory process involving 3^rd^ brachial space.

He subsequently underwent excision biopsy of the cystic lesions. The histopathological findings showed papillary thyroid carcinoma which is positive TTF-1, thyroglobulin, CK19 expression and negative reaction towards Napsin A. An impression then was given as PTC with occult primary from thyroid gland cannot be completely excluded.

A month later, the patient was then undergoing total thyroidectomy and modified left radical neck dissection. The thyroid gland is of normal size with a small white nodule noted at left upper pole subcapsular region (4 x 3 mm), pale lesion at mid right lobe (4 x 4 mm), and pyramidal lobe (2 x 1 mm), in keeping with multifocal papillary microcarcinoma. The cervical lymph nodes showed metastatic papillary thyroid carcinoma involving 8 out of 19 nodes. He is currently undergoing radioactive iodine ablation.

## 3. Discussion

Most of the lateral neck cysts are benign such as branchial cleft cysts, dermoid cysts, teratoma, epidermoid cysts, and cystic hygromas. In 90% of patients in the young adult population cervical neck cysts were proven to be benign [7]. Conversely, PTC presented as a lateral cystic neck mass without palpable lesion in the thyroid gland is rare. It may arise from thyroid primary which underwent cystic degeneration or true malignant transformation of ectopic thyroid tissue [8]. The latter condition is extremely rare. In our case report, we described two cases of PTC on the lateral neck cyst. These cases almost have similar clinical courses and radiological as well as histopathological findings. Ultrasonography and CT scan of neck region for both cases revealed no evidence of lesion in the thyroid gland. However, the patient in Case 2 was subjected for total thyroidectomy and histopathological results showed the origin of primary tumour.

Cervical lymph node metastasis of PTC can mimic benign cervical cyst clinically and radiologically when it underwent cystic degeneration. Metastasis to cervical lymph nodes could be the first presentation without identifiable primary tumour in thyroid glands [9]. Cervical lymph node metastasis in patient with occult thyroid carcinoma has occurred in 30% of all cases of thyroid carcinoma and these metastatic lymph nodes usually presents as palpable masses in the anterior and lateral aspect of the neck [2]. Clinically, it might be difficult to make a differentiation at initial presentation between a cystic lymph nodes metastatic from an impalpable occult PTC and a benign cervical cyst.

Ultrasonography and nuclear medicine are an important diagnostic tool in the assessment of thyroid nodules. However, misdiagnosis on ultrasound may occurred due to it being an operator dependent procedure which requires growing experience and skill [10]. Furthermore, a very small thyroid nodule may not be significantly detected on ultrasound. Due to limitations, of ultrasound imaging for a small thyroid lesion, the radionuclide thyroid scan helps in arriving to definite diagnosis. In Case 2, the thyroidectomy specimen examination revealed that the size of the nodules is too small. Thus, it is not impossible that some of the PTC cases may go undetected, despite modern imaging modalities and high-resolution ultrasound. These are due to difficulty visualizing small lesions or benign ultrasonographic appearance [11].

American Thyroid Association (ATA) 2015 stated that the prompt treatment for PTC is the removal of the primary tumour together with the clinically significant lymph nodes metastasis [12]. These steps are important in order to determine the outcome and recurrence of the disease. Thus, early detection of thyroid carcinoma in patient with lateral neck cyst is crucial because the course of treatment in such cases is very different from those with benign cervical cyst.

In conclusion, the incident of the PTC on the lateral neck is not uncommon. Surprisingly, until now the reported cases are increasing in trend. Thus, thorough investigations including total thyroidectomy are warranted in all cases of PTC on the lateral neck cyst. This practice is to ensure that PTC can be detected earlier because it has a very good prognosis if treated at initial stage.
